# Clearing the Transcription Hurdle in Dialect Corpus Building: The Corpus of Southern Dutch Dialects as Case Study

**DOI:** 10.3389/frai.2020.00010

**Published:** 2020-04-15

**Authors:** Anne-Sophie Ghyselen, Anne Breitbarth, Melissa Farasyn, Jacques Van Keymeulen, Arjan van Hessen

**Affiliations:** ^1^Department of Linguistics, Ghent University, Ghent, Belgium; ^2^Variaties VZW, Umbrella Organisation for Dialects and Oral Heritage, Brussels, Belgium; ^3^Human Media Interaction, Faculty of Electrical Engineering, Mathematics & Computer Science, University of Twente, Enschede, Netherlands

**Keywords:** dialect, transcription, corpus research, ASR, respeaking, forced alignment, dutch, Flanders

## Abstract

This paper discusses how the transcription hurdle in dialect corpus building can be cleared. While corpus analysis has strongly gained in popularity in linguistic research, dialect corpora are still relatively scarce. This scarcity can be attributed to several factors, one of which is the challenging nature of transcribing dialects, given a lack of both orthographic norms for many dialects and speech technological tools trained on dialect data. This paper addresses the questions (i) how dialects can be transcribed efficiently and (ii) whether speech technological tools can lighten the transcription work. These questions are tackled using the Southern Dutch dialects (SDDs) as case study, for which the usefulness of automatic speech recognition (ASR), respeaking, and forced alignment is considered. Tests with these tools indicate that dialects still constitute a major speech technological challenge. In the case of the SDDs, the decision was made to use speech technology only for the word-level segmentation of the audio files, as the transcription itself could not be sped up by ASR tools. The discussion does however indicate that the usefulness of ASR and other related tools for a dialect corpus project is strongly determined by the sound quality of the dialect recordings, the availability of statistical dialect-specific models, the degree of linguistic differentiation between the dialects and the standard language, and the goals the transcripts have to serve.

## Introduction

In the history of dialectological research, corpus research has long been scarce. Dialect atlases and dictionaries traditionally build on survey data and/or introspective data (native speaker judgments), rather than on databases of spontaneous speech samples. The reasons for the popularity of these survey and introspective data are quite obvious: (1) on the basis of elicitation and introspection, the diverse aspects of a dialect's lexicon, phonology, morphology, and/or syntax can be studied more systematically, by restricting the focus to controlled conditions (cf. Cornips and Poletto, 2005), and (2) the collection and analysis of elicited/introspective data are also less time-consuming than dialect corpus building and analysis. The restriction to predefined conditions, however, while making research efficient, replicable, and comparable, is also a major limitation. Dialect corpus research has clear advantages over elicited data here: analyzing spontaneous speech not only allows insight into the functional strength of dialect features in real life but also makes possible a more thorough study of dialect phenomena conditioned by discourse or register, phenomena that might remain unnoticed in survey data. Not in the least, it allows for the serendipitous discovery of phenomena that previously escaped attention and are therefore not considered in the construction of surveys.

In usage-based approaches (Kemmer and Barlow, 2000; Bybee, 2010) as much as in more formalist (especially historical) research (cf. contributions in Jonas et al., 2011; and Mathieu and Truswell, 2017), corpus analysis has strongly gained in popularity (cf. Szmrecsanyi and Anderwald, 2018), as frequency data are a way to uncover/reconstruct the linguistic knowledge underlying the usage, and to study contextual factors affecting it. This development is also fostered by the availability of Automated Speech Recognition (ASR) tools and Natural Language Processing (NLP) software facilitating automated audio and text annotation. Remarkably, however, *dialect* corpora are still relatively scarce, especially when the term ‘dialect’ is interpreted in the ‘traditional’ sense as regionally determined language varieties that differ at multiple structural levels—phonetic, phonological, morphological, lexical, syntactic, and/or semantic—from other dialects and the ‘overarching’ standard language (cf. Trudgill, 1999, p. 5; Boberg et al., 2018, p. 4–5).^1^ A number of factors account for this scarcity. First, dialects are generally spoken in informal/private domains, making it challenging to collect samples of these language varieties. In contrast to standard language corpora, one cannot partly rely on ‘public’ speech settings, such as news broadcasts, TV shows, or parliament debates for data collection. Secondly, as ASR and NLP tools are usually trained on standard language data, it can be quite challenging to apply these tools to dialect data. As such, transcribing or annotating dialect data usually requires more manual work than standard language data (or regionally accented language use). Even when disregarding the functioning of ASR and NLP tools, the process of putting speech to text—the first essential step in the building of speech corpora—is much more challenging for dialect recordings than for standard languages, as for many dialects orthographic norms are not available.

Interestingly, the transcription problem in dialect corpus research has received little scientific attention, which is strange given the increased interest in transcript-based research the last decades. In this paper, we aim at filling this methodological gap by addressing the questions (i) how dialects can be transcribed efficiently and (ii) whether NLP tools can lighten the transcription work. These questions will be tackled using the Southern Dutch dialects (SDDs) as case study, i.e., the dialects spoken in (i) Dutch-speaking Belgium, (ii) the three southern provinces of the Netherlands (Limburg, Noord-Brabant, and Zeeland), and (iii) the Flemish-speaking dialect region in France.^2^ The discussion is based on the results of a pilot project laying the foundations for a large-scale Corpus of SDDs. The pilot project, which focused on the dialect collection *Stemmen uit het Verleden* (‘Voices from the past,’ Ghent University)^3^, aimed at developing a transcription protocol and an annotation pipeline and establishing benchmarks for the transcription, correction, and annotation of Dutch dialect recordings.

## Toward a Corpus of SDDs

The SDDs have been shown to have a number of striking typological characteristics (see, e.g., De Vogelaer, 2008; De Schutter, 2009; Taeldeman and De Wulf, 2010; Swanenberg and van Hout, 2013; Breitbarth and Haegeman, 2014), with dialects diverging phonologically, morphologically, syntactically, and lexically from both the Dutch standard language^4^ and each other. In the light of the so-called “delayed” standardization process in Flanders (Vandekerckhove, 2009, p. 75), dialect leveling processes have set in quite late (compared to other European speech communities), and hence, dialects still often vary from village to village or from city to city. This dialect diversity is interesting for language-historical research, as the SDDs form a missing link in the language history since Middle Dutch: the SDDs played only a minor role in the standardization processes mainly going out from the northern provinces since the seventeenth century, and were hardly affected by them (cf. Willemyns, 2003).

Much of the more recent research into the SDDs is either based on the big dialect atlases of Dutch, i.e., the *Fonologische Atlas van de Nederlandse Dialecten* (FAND, 1998/2000/2005; ‘Phonological Atlas of the Dutch Dialects’), the *Morfologische Atlas van de Nederlandse Dialecten* (MAND, 2005/2009; ‘Morphological Atlas of the Dutch Dialects’), and the *Syntactische Atlas van de Nederlandse Dialecten* (SAND, 2005/2008; ‘Syntactic Atlas of the Dutch Dialects’), which are based on elicited data, or on introspective data (native speaker judgments). As already discussed in the *Introduction*, there are a number of problems with these methods when it comes to linguistic research, especially into the syntax of Flemish dialects. For example: contrary to Standard Dutch, some SDDs can have the verb as the third constituent in the clause [cf. (1)] instead of the second one, if the clause is introduced by an adverbial element (Haegeman and Greco, 2018; Lybaert et al., 2019).

1. Met zulk weer je kunt niet veel doen.
                                         (SAND sentence 359)
  with such weather you can NEG much do
  “With such weather, you cannot do much.”

Data such as (1) are underreported in the *Syntactische Atlas van de Nederlandse Dialecten* (SAND, 2005/2008), as many types of these so-called V3 constructions are only realized in very specific pragmatic contexts (e.g., to indicate that something comes as a surprise) and are hence difficult to elicit in a survey. Indeed, for several locations, the SAND fieldworkers observe that even though rejected by the informants, the pattern is attested in their spontaneous speech, which the notes of the fieldworkers acknowledge.^5^ This is only one example of a phenomenon that would benefit from being studied on the basis of a corpus of spontaneously spoken dialect (complementary to survey data analysis).

**Figure 1:**
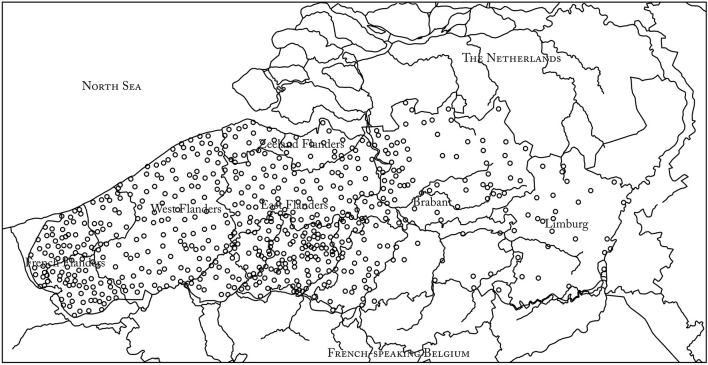
Regional spread of the dialect recordings of the collection “Stemmen uit het Verleden.”

In the 1960s and 1970s, dialectologists at Ghent University made 783 tape recordings of 45 min on average (in total about 700 h) in 550 locations (cf. Figure 1) in the Dutch-speaking provinces in Belgium, Zeeland Flanders (Netherlands), and French Flanders (France). Their goal was to build a corpus for dialect research. The recorded speakers—often practitioners of an occupation considered vanishing or ‘lost’ at the time of recording—are born in the late nineteenth and early twentieth centuries (the oldest speaker was born in 1871) and are almost always monolingual dialect speakers, and because most of the speakers have received only minimal formal education, their speech is hardly influenced by the Dutch standard language. The speakers were generally interviewed by a fellow villager in the local dialect to avoid adaptation to the language of the interviewer. The topics of the conversations were free; in contrast to, for instance, the interviews for the SAND or the *Syntax hessischer Dialekte* (SyHD, Fleischer et al., 2015), the aim was not to elicit specific linguistic constructions. In general, the speakers narrate about their life, profession, and the sociocultural changes they witnessed during their lifetime. This makes the material, which has become known under the name *Stemmen uit het Verleden* (‘Voices from the past’), valuable not only for linguistic purposes, but also for (oral–) historical and cultural–historical reasons.

The collection of dialect recordings constitutes a valuable data source both for large-scale phonological, morphological, lexical, and syntactic research and for the study of specific phenomena that are mainly restricted to spontaneous speech, and which therefore resist elicitation. Because the speakers recorded were born around the turn of the twentieth century, and hence acquired language about 100–120 years ago, these recordings already represent a historical stage of the language. Additionally, the tapes contain accounts of oral history that may provide valuable information on, e.g., the events around the World Wars. Moreover, the recordings constitute a treasure trove of cultural heritage, such as lost professions and customs.

**Figure 2:**
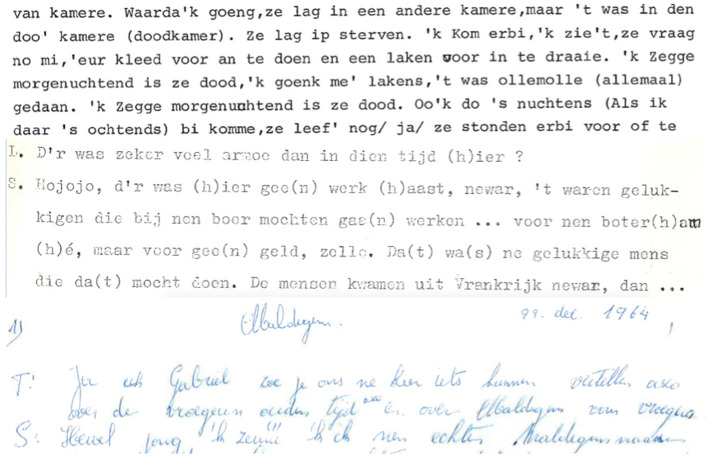
Excerpts from existing transcriptions for recordings in Torhout, Wichelen, and Maldegem, respectively.

The accessibility of the recorded dialect data is undeniably invaluable for linguistic and historical research. However, the vast collection of data can currently hardly be used for linguistic or historical research, as the material is not digitally searchable for word forms (allowing one to make concordances of keywords in context), let alone for syntactic patterns and constructions. Thanks to various projects such as *Stemmen uit het verleden* (“Voices from the past”; see www.dialectloket.be), the tapes have been digitized and safeguarded for posterity. Nevertheless, various hurdles have to be overcome to make the material fully accessible for researchers. Firstly, only 318 of the 783 recordings have been transcribed. These transcriptions were generally made in the 1960s and 1970s by students writing a dissertation on dialect syntax. Secondly, the transcriptions that exist (i) are only available electronically in the form of scans (i.e., image files) of the original typewritten or even handwritten texts, (ii) often contain many mistakes, (iii) are not time-aligned to the audio (cf. infra), and (iv) are heterogeneous in the way the dialect has been transcribed. This heterogeneity can be attributed to the fact that there is hardly a writing tradition in the dialect—dialects have been passed on orally from generation to generation—and that only a brief transcription guideline was provided. Figure 2 illustrates the heterogeneity by means of three excerpts from existing transcriptions, two in typoscript and one in handwriting. Whereas in the first and third excerpt, non-standard Dutch vocalism is rendered in a kind of ‘eye dialect’ (e.g., in excerpt 1: *ip* and *ollemolle* instead of standard Dutch *op* ‘on’ and *allemaal* ‘all’; in excerpt 3: *zeune* instead of standard Dutch *zijn* ‘be’), non-standard vocalism has been standardized in the transcription from Wichelen. The dialectal vowel in *gaan* (‘go’), which is pronounced as [ɔˑ] in the recording from Wichelen, is for instance not rendered in the transcription. A similar heterogeneity can be seen in the way the deletion of initial or final consonants is marked: in the first excerpt, apostrophes are used (e.g., *me'* for standard Dutch *met* ‘with’), while in the second, the deleted consonant is reconstructed between brackets [e.g., *da(t)* for standard Dutch *dat* (‘that’)]. These are only some examples of the heterogeneity in the existing dialect transcriptions. Bearing in mind the currently rapidly advancing dialect loss across Flanders (Vandekerckhove, 2009; Ghyselen and Van Keymeulen, 2014), there is a real risk that soon there will not be any speakers able to understand and hence to (help) transcribe them. In order to make this unique collection of dialect data present at Ghent University accessible for fundamental research, their transcription and linguistic annotation is therefore of high priority. Achieving these two goals is the core of the project *Gesproken Corpus van de zuidelijk-Nederlandse Dialecten* (GCND, Spoken Corpus of the SDDs).

## Protocol Requirements

Transcribing is a process of data reduction: some elements of the speech signal are visualized in the transcript; others are ignored. A transcript is hence always a research construct (Jenks, 2011, p. 11), the result of numerous decisions on which elements to graphically render and which not. As dialect corpus research requires transcripts to be “as reliable, faithful, and internally consistent as possible” (Szmrecsanyi and Anderwald, 2018, p. 302), it is of utmost importance that a detailed protocol is developed, which ensures that all transcribers take the same or rather similar decisions in the data reduction process.

In devising a protocol, it is vital to keep the purposes of the collection in mind, which in the case of the GCND are diverse. On the one hand, the transcripts in our corpus have to cater to the needs of linguists interested in the diverse aspects of the dialect system. The main purpose of the GCND is to provide a database for both corpus-based and corpus-driven research (Biber, 2009) on the syntax of the Dutch dialects, as syntactic patterns—especially optional constructions (Cornips and Poletto, 2005, p. 955)—are known to be especially difficult to study via elicitation. However, the corpus should ideally of course also allow morphological, lexical, and phonological/phonetic research, e.g., dialectometric research measuring the phonetic distance between dialects (cf. Heeringa, 2004; Nerbonne and Heeringa, 2010). In this context, (i) a high transcription accuracy and consistency is needed and (ii) transcribers cannot simply standardize non-standard words, pronunciations, or constructions, as this is exactly what dialectologists are interested in. On the other hand, the transcripts should also be accessible for historians, ethnologists, or laymen interested in the content of the tapes. For this reason, the texts should also be readable to those not thoroughly familiar with phonetic alphabets or all the specificities of the local dialects. A further consideration in choosing a transcription protocol is that the transcripts should allow NLP tools to automatically annotate the texts with POS tags^6^ and syntactic parsing information and that such tools are typically trained on standard language resources and hence benefit from transcriptions close to standard language norms.

To allow phonetic or phonological research, the dialect transcriptions should preserve as much phonetic detail as possible. However, manual phonetic transcriptions are—more than other types of transcriptions—very sensitive to transcriber effects, and hence pose a problem for transcript consistency. Bailey et al. (2005) discuss how, even after careful phonetic training of transcribers, the phonetic transcriptions needed for the *Linguistic Atlas of the Middle and South Atlantic States* (LAMSAS) and the *Linguistic Atlas of the Gulf States* (LAGS) were clearly subject to transcriber effects, due to “(1) conceptual differences regarding the phonetic status of particular sounds (e.g., offglides of diphthongs) and how they should be transcribed, (2) normative differences regarding the phonetic values of particular symbols, and (3) changing scribal practices as transcribers discover the importance of phonetic details that they had previously overlooked” (Bailey et al., 2005, 3). Phonetic transcription is also much more time- and hence also budget-consuming than orthographic transcription. In the context of the GCND, for which a large number of transcribers collaborate on the transcription of 700 h of very diverse spoken dialects, it was quickly decided that manual phonetic transcription was simply not feasible. As an alternative, time and effort was invested to link all layers (or tiers) of annotations to time codes in the audio, thereby ensuring that researchers interested in phonetic detail can easily consult the passages of the audio relevant for their research purposes and provide phonetic annotations themselves. Below (see section ‘Forced alignment for automatic segmentation and phonetic transcription’), we also investigate the possibility of automatic phonetic transcription via forced alignment (FA).

Leaving the option of manual phonetic transcription aside, the question arises how non-standard pronunciations, lexical items, and syntactic structures—in the absence of writing norms for Dutch dialects—should be rendered in the Latin alphabet. In addressing this question, a difficult balance has to be struck between faithfulness to the original dialect on the one hand and regularization to guarantee consistency, searchability, and accessibility for non-linguists on the other hand. Given that interoperability and sustainability/reusability are important requirements in the year 2019 when collecting and annotating data (cf. the philosophy of CLARIN, the European Research Infrastructure for Language Resources and Technology; De Jong et al., 2018), it is interesting to consider how this balancing act has been performed in earlier variationist research.

## Building on Existing Standards

The approaches chosen in existing projects transcribing non-standard or dialect speech range from almost entirely using standard orthography to layered transcriptions differentiating phonological variation and standard orthography. The COSER^7^ and PRESEEA^8^ corpora of dialectal and spoken Spanish, for instance, use one layer of orthographic transcription, which also represents a number of divergences from the standard language orthographically. In COSER transcripts, non-standard stress positions and omissions and additions of phonological segments are systematically rendered orthographically. The FOLK corpus of spoken (near-standard) German (Schmidt, 2016)^9^ and the CORDIAL-SIN corpus of Portuguese dialects^10^ in principle transcribe orthographically in one layer except for some individual words. FOLK is transcribed in a modified orthography (‘eye dialect,’ cf. Schmidt, 2016, p. 119) following the GAT2-standard (Selting et al., 2009, also used in the research project *Deutsch in Österreich*^11^). The corpus, however, also provides normalized forms for divergent items as word-level tags to the original transcription. This normalized transcription is the input to further NLP processing. The transcription in CORDIAL-SIN^12^ uses the standard orthography even in the case of regionally divergent phonology, except in cases that are considered as potentially relevant for future (morpho)syntactic analysis. In such cases, divergent phonetic realizations, contractions, and truncations are marked in the same layer by stating the divergent form and the standard form next to each other, e.g., deu-{PH|li=lhe} for (standard Portuguese) *deu-lhe* ‘(he) gave him’ for phonetic variation in the pronunciation of the clitic *lhe*, or {IP|'pεɾɐ=espera} for the truncation of the initial part of the word *espera* ‘wait.IMPV.’ For further NLP processing (morphological tagging and syntactic parsing), only the normalized form is used, which is produced automatically from the original transcription by replacing, e.g., {IP|'pεɾɐ=espera} by *espera*. This normalized form is stored in a separate file (ASCII and .pdf). The last possibility, fully transcribing in two layers, with one layer representing the original dialect and one ‘translating’ the dialect into standard orthography, is not used by existing spoken language corpora as far as we are aware. This is presumably due to a large degree of overlap between the produced dialect strings and the standard language. Such overlap is however somewhat problematic for the SDDs, as there is a high degree of phonological, lexical, morphological, and syntactic divergence between dialects and standards, which complicates a procedure marking all forms diverging from the standard language with individual tags.

In the Dutch language area, there are no digital corpora of spontaneously spoken dialect yet. The large-scale Corpus of Spoken Dutch (*Corpus Gesproken Nederlands* or *CGN*, Oostdijk, 2000)—containing approximately 9 million words—focuses on (intended) standard language, and hence its transcription protocol is not geared toward dialect research.^13^ There is however a rich tradition of dialect study in the Dutch language area (cf. Goossens and Van Keymeulen, 2006) and, as such, there are already conventions for dialect orthography to be built upon. For the GCND protocol, Barbiers and Vanden Wyngaerd (2001) was taken as point of departure, who describe the transcription guidelines used for the *Syntactic Atlas of Dutch Dialects*.^14^ For this SAND project, transcriptions were made of questionnaires—asking for the judgment and/or translation of some 150 test sentences—conducted orally (fieldwork and telephone) between 2000 and 2005 in about 300 locations across The Netherlands, Belgium, and a small part of north-west France. The protocol was devised with syntactic purposes in mind, and hence opts for strong standardization of non-standard pronunciation in content words, whereas non-standard functional elements (inflection, pronouns, articles, auxiliaries, etc.) and syntactic structures (word order, double negation, and extra complementizers) are transcribed as closely to the dialect as possible. For the GCND protocol, a comparable approach was adopted:

- Phonological variations of content words that also exist in the standard language are spelled according to official standard language orthography (as established by the Dutch Language Union in the *Woordenlijst Nederlandse Taal*).^15^ If a speaker for instance pronounces the standard language word *steen* ([steːn] ‘stone’) with a diphthong (e.g., [stiˑən]), we write *steen*; for reasons of intertranscriber consistency and readability, these non-standard pronunciations are not rendered in some kind of ‘eye dialect’ (we do not write *stieën*).
- Content words that do not have an equivalent in the standard language are written down following the principles of Standard Dutch spelling as closely as possible. The word [lɑtəstoˑərs] for instance (‘roll-down shutters’) is written down as *lattestoors*. Non-standard words are *not* translated into a standard Dutch alternative (such as *rolluik*), as (i) these non-standard lexemes are of interest to dialectologists and (ii) the precise translation of these dialect words is often open to debate. If the non-standard words have already been included in an existing dialect dictionary (e.g., www.e-wvd.be for the Flemish dialects, www.e-wbd.nl for the Brabantic dialects, and www.e-wld.nl for the Limburgian dialect), transcribers adopt the dictionary spelling. To guarantee transcriber consistency, a logbook of non-standard lexemes and their spelling is shared among transcribers.
- Function words (inflection, adpositions, auxiliaries, determiners, negation particles, conjunctions, and pronouns) are transcribed as close to the dialect as possible, with an orthographic rendering of deletions and insertions of consonants (cf. Moreno et al., 2016 on the Spanish COSER corpus). If a speaker pronounces *wat* (‘what’) without final [t], the deletion is also written down (*wa*). Vocalic changes with functional value (e.g., *vuut* ‘foot.pl,’ standard Dutch *voeten* ‘feet,’ with the umlaut marking the plural) are also transcribed, following standard Dutch orthographic rules as accurately as possible. Regular changes in the vocalism [e.g., the pronunciation of standard Dutch [a:] as [ɔː] in for instance *maar* (‘but’)] are however not transcribed, but rendered in standard Dutch spelling, as trying to consider all these phenomena would compromise the consistency among transcribers.
- Non-standard clitics [e.g., *tkind* for standard Dutch *het kind* (‘the child’)] are written down as clusters of elements, using hashtags to mark—intuitively—the different elements part of the cluster (e.g., *t#kind*). This ‘hashtag analysis’ is not a fixed fact, but has the status of a ‘first guess’ (cf. Barbiers and Vanden Wyngaerd, 2001, p. 6).
- Non-standard syntactic constructions (e.g., with subject duplication or alternative word orders) are transcribed as close to the dialect as possible.

To cater to the needs of non-linguists intending to search the content of the tapes and to facilitate the functioning of NLP tools—which are mainly trained on standard language data—an extra transcription layer is added in the GCND corpus, a layer in which function words are standardized (*gunder* or *gider*, e.g., are written down as *jullie* ‘you [plural]’) and clitics are separated into their component parts (e.g., *t#kind* is written down as *het kind* ‘the child’). In this standardized layer, non-standard lexemes and non-standard constructions are preserved, as it is often unclear what the standard language equivalents for these words and constructions should be. All layers of transcription are time-aligned to the audio using ELAN (Max Planck Institute for Psycholinguistics, cf. Brugman and Russel, 2004).^16^ Example sentence (2) showcases the different principles outlined in GCND protocol.

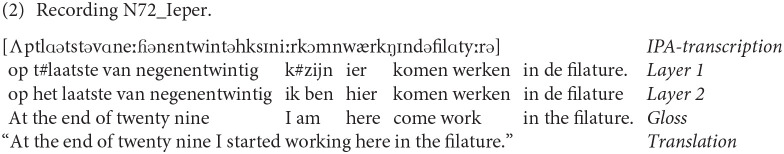



The first layer in (2) stays close to the original dialect, orthographically rendering:

- non-standard words, here the dialect word *filature* for standard Dutch *spinnerij* (‘filature’),
- non-standard morphology (e.g., *zijn* for standard Dutch *ben* ‘am’),
- clitics (e.g., *t#laatste* for standard Dutch *het laatste* ‘the end’),
- the insertion or deletion of consonants in function words (e.g., *ier* for standard Dutch *hier* ‘here’), and
- non-standard syntax [cf. word order adverbial *(op t#laatste van negenentwintig)* + subject *(k)* + conjugated verb *(zijn)* instead of Standard Dutch adverbial + conjugated verb + subject in main clauses].

Non-standard variations of content words that also exist in the standard language are however standardized. We, for instance, write *negen* ‘nine’ and not *nehen*, even though the speaker clearly laryngalizes the fricative [ɤ]. In the second layer, the non-standard lexeme *filature* and non-standard syntactic constructions (lack of inversion after the adverbial phrase) are preserved, but clitics are written down as clusters of elements (*t#laatste* > *het laatste*), deleted consonants are ‘restored’ (*ier* > *hier*), and the morphology is standardized (*k#zijn* > *ik ben*).

## Speech Technology to the Rescue?

The transcription procedure outlined above is—when performed manually—very time-intensive and therefore expensive. Transcription speeds for our data range from 67 s/h for a beginning transcriber to 120 s/h for an experienced one. The question arises whether speech technology can speed up the process. In what follows, we review a number of methods that can potentially accelerate the transcription and/or alignment process: automatic speech recognition (ASR, section Automatic speech recognition), respeaking, and forced alignment (FA).

### Automatic Speech Recognition

In the last few decades, significant headway has been made in ASR. ASR analyzes the sound spectrum of the input speech and tries to determine—on the basis of a language or even dialect specific *acoustic model*—which phonemes could correspond to the input spectra. An acoustic model contains statistical representations for each phoneme in a language, created from a set of audio recordings and their corresponding transcripts. Next, the obtained set of phonemes is used to estimate via a (dedicated) *language model* the words that could have been spoken. A language model is a statistical model that represents the probabilities of words and phrases in a specific language. The result of this estimation process is a set of words with their start time, duration, and recognition probability. Modern ASR engines like the KALDI and Google recognizers can recognize 256K different words.

ASR has many applications. It is for instance increasingly used for spoken document retrieval, as illustrated by the FAME! Project (Frisian Audio Mining Enterprise). This project developed an ASR system for Frisian–Dutch code-switching speech, as extracted from the archives of a local broadcaster. The goal of the system was to allow automatically retrieving relevant items from a large collection of news broadcasts, in response to user-specified text queries (Yilmaz et al., 2018, p. 12). Similarly, Van Den Heuvel et al. (2012) report applying ASR to disclose—via keyword retrieval−250 interviews with veterans of Dutch conflicts and military missions. ASR also has applications in reporting. Kawahara (2012) discusses the development of a speaker-independent ASR system for transcribing plenary and committee meetings of the Japanese Parliament. This system is said to consistently produce accuracy levels of at least 85%. The automatically generated transcripts are then further processed by parliamentary reporters. The usefulness of ASR for reporting purposes, however, strongly depends on the language under study. An innovation project carried out in Flanders in 2017–2018 led to the conclusion that the state of speech-to-text technology for Dutch was insufficient at the time to be useful for reporting debates of the Flemish Parliament, as it did not increase, but rather reduced reporting efficiency.^17^

In linguistic research, ASR remains little used for full automatic transcription. There are, however, examples of successful application. Michaud et al. (2018) for instance describe how ASR advanced the study of Yongning Na, a Sino-Tibetan language of Southwest China. Of the 14 h of speech the authors recorded during fieldwork, 5.5 h (both narratives and morphotonology elicitation sessions) were transcribed by hand. Subsequently, an ASR transcription tool was trained on these transcribed materials, in order to perform phoneme recognition on the remaining untranscribed audio files. The error rate of the resulting transcriptions proved low, about 17%. According to the authors, the automatic transcriptions reduced the manual effort required for creating transcripts and allowed new insights that might not have been gained by the linguist alone.

Via user-friendly interfaces building on neural network models (e.g., Cloud Speech-to-Text by Google), even computational laymen can now attempt to convert audio to text automatically. A quick test in Google Cloud Speech-to-Text on 129 words of intended Standard Dutch, as spoken by a highly educated West Flemish speaker in a standard language test (cf. Ghyselen, 2016) yields a fine Word Error Rate (WER)^18^ of only 7%. As ASR systems can also add time codes in the transcription—useful to align the text to the original audio—ASR offers interesting opportunities for speech corpus building.

However, many dialects—such as the Southern Dutch ones—must be considered ‘low resource languages,’ i.e., languages for which few tools and/or resources are available. This constitutes a major challenge for the application of ASR. While acoustic and language models for Netherlandic Dutch and Belgian Dutch have been developed (cf. https://www.spraak.org and https://spraaktechnologie.org), these were mainly trained on standard language and on regionally colored speech, which is much closer to the standard norm than the dialects in our data collection. Generally, tools trained on standard language underperform on non-standard data. While the intended standard Dutch sample of the highly educated West Flemish speaker discussed above yielded a WER of only 7% in Google Cloud Speech-to-Text (cf. Ghyselen, 2016), the WER increased to 66% in a test using 164 words of a spontaneous interview by the same West Flemish speaker. This is high, considering that the language used in the interview is not fully fledged dialect, but only diverges in some pronunciation features from the official standard language (especially h-dropping and t-dropping) and that the recording quality was high. Note that the option ‘Netherlandic Dutch’ had to be used, as ‘Belgian Dutch’ was not available. The low-resource problem is—as can be expected—only exacerbated with dialect data. Tested on a dialect recording from the *Voices of the Past* collection^19^, Google Cloud Speech-to-Text obtains a WER of 90%. A comparison of the reference transcription in (3) with the automatic transcription in Google Cloud Speech-to-Text (option: Netherlandic Dutch) in (4) illustrates how ASR is at present not helpful as a tool to speed up the transcription process in the GCND project.

(3) k#e vijf jaar in Tourcoing ewrocht in e fabrieke. van negen…uh van drieëntwintig tot negenentwintig. en in ne…op t#laatste van negenentwintig k#zijn ier komen werken in de filature. vierendertig jaar. en k#e moeten twee jaar eerder mijn pensioen nemen. omda#k epakt waren aan mijn harte. en ezo k#zijn nu gepensioneerd. k#zijn nu tweeënzestig nu nieuwjaar. twee dagen voor nieuwjaar zij#k tweeënzestig. ja en k#zijn al elf jaar mijn man kwijt wi. awel ja#k. ja k#e maar een zoone.
*I have worked for five years in Tourcoing in a factory. from nine… uh from twenty-three to twenty-nine. and in… in the end from twenty-nine I have come here to work in the filature. thirty-four years. and I have had to retire two years earlier. because I had heart problems. and as such I am retired now. I'll turn sixty-two at new year. two days before New Year I am sixty-two. yes and I have lost my husband for eleven years already. yes I have. yes. I have only one son*.
(4) fabrieken van 23 tot 29 van 29,34 jaar omdat tweedehands *factories from 23 to 29 from 29.34 years because second-hand*

The ASR tool of the BASWebServices of the Ludwig Maximilian University of Munich^20^ performed equally poorly (WER = 95%), with the following output:

(5) koel je nou vooral ten fabrieken van van drieëntwintig tot negenentwintig van negenentwintig vierendertig jaar heb ik haar mond nu twee jaar om daskapan kwamen bij maar dat is ook zien in een gepassioneerd twee dan voor een nieuwe hadden ja die arme man with we elkaar Morrison
*cool you now especially at factories of of twenty-three to twenty-nine from twenty-nine thirty-four years have I her mouth now two years for daskapan*^21^ *came at but this is also see in a passionate two then for a new had yes that poor man with we each other Morrison*.

Of course, ASR tools (including the acoustic and language models) can be adjusted/retrained on new data to cater to the needs of dialectologists, but currently, no suitable tools exist. Furthermore, the retraining of such tools typically requires large amounts of already transcribed text from all dialects to be efficient.

In deliberating the usefulness of ASR investments (e.g., developing dialect-specific acoustic and language models) in a dialect corpus project, there are different factors to consider. A first one is the sound quality of the audio collection: recordings with background noise, much overlapping speech and/or a large variance in recording settings (distance from microphone etc.), present a bigger challenge for ASR systems. Michaud et al. (2018, p. 396) point out that the high audio quality of their recordings of Yongning Na speech is an important part of the reason why the automatic transcription yielded good results. The authors stress that for low-resource languages, it is highly important that the pronunciation is clear and the audio signal is clean. In the case of our dataset, the recordings were made in the 1960s and 1970s in 550 locations (often private homes of dialect speakers, with barking dogs, ticking clocks, or vehicles passing by as background noise) with reel-to-reel tape recorders and often multiple speakers per recording. The acoustic properties as such differ from recording to recording, which implies serious challenges for ASR systems.

Secondly, the performance of ASR tools strongly depends on the degree of linguistic differentiation between the dialects and standard language. As explained above, the SDD systems diverge significantly phonologically, morphologically, syntactically, and lexically from the Dutch standard language, which explains why tools developed for Netherlandic (Standard) Dutch perform so poorly on SDD recordings. It is not easy to extend the existing tools for non-standard speech, as this requires a significantly large training set of transcribed dialect, which is not available for the Dutch dialects. In the last few years, the dictionaries of the Flemish, Brabantic, and Limburgian dialects have become available online (cf. e-wvd.be, e-wbd.nl, and e-wld.nl, respectively), which offers opportunities for ASR systems, but the keywords in these dictionaries are ‘standardized’^22^ —given the lack of orthographic norms for the dialects (cf. section ‘Toward a corpus of Southern Dutch Dialects’)—and as such, the ASR systems will need enough training data to link the acoustic realization of non-standard words to the keywords in the dictionaries. An important issue is also the diversity among dialects: global tools simultaneously trained on many dialects have been reported to “fail to generalize well for any of them,” as a consequence of which state-of-the-art speech recognition systems, including that of Google, prefer building a different recognizer per “dialect” (Elfeky et al., 2018, p. 2). In the case of the SDDs, the diversity is so large—with four big dialect areas that are internally also very diverse morphologically, phonologically, syntactically, and lexically—that multiple recognition systems should be built, implying serious time and financial investments.

A third factor to bear in mind is the goals the ASR transcripts have to serve, as this determines the transcript accuracy needed. For example: the FAME! Project already introduced above obtained WERs ranging from 32 to 33% (Yilmaz et al., 2018, p. 18–19), which is a satisfying result when the goal of the transcripts is to make the broadcast archive more searchable content-wise. Ordelman et al. (2007, p. 214) mention a WER of 50% as baseline for spoken document retrieval. However, when the goal of a project is to facilitate linguistic research, a higher transcription accuracy is needed. If the researcher has to correct 1 out of 2 words manually after implementing ASR, he might as well not lose time (and money) on ASR and transcribe the speech manually from the beginning. A tough question to answer is what ballpark area WERs have to be in for ASR (or another speech technological tool) to become a viable option in linguistic research, e.g., to provide a first draft. Human transcribers are said to have error rates ranging between 3 and 10%, depending on the type of input speech and the time spent on the transcription (Stolcke and Droppo, 2017, p. 137–138). In the context of the GCND project, a comparison of student transcriptions for four recordings of four different dialect areas with the final equivalent as corrected by both an older speaker of the recorded dialect and the project coordinator yields an average WER of 3% (lowest = 0.4%, highest = 6.4%). This WER is difficult—not to say impossible—to equal with ASR (at least when it concerns non-standard speech), but there is still the option of first creating a draft transcript using ASR and then manually correcting it. Ranchal et al. (2013) report the results of such an approach to transcribe lectures taught in English. They obtained—after voice profile training—WERs of 22% for the automated first transcript. The manual correction of these automated transcripts is said to take 4 h per hour of lecture audio (Ranchal et al., 2013, p. 306–307), which still is a lot, given that the researchers also invested time in the ASR development and voice profile training. It hence seems logical to assume that with WERs of over 30%, it is time and budget friendlier to transcribe the recordings manually from the start.

Considering the issues discussed above in the context of the GCND, the decision was made not to invest in ASR development, given (i) the very diverse acoustic properties of the recordings, (ii) the current lack of training data, (iii) the diversity among the SDDs and the large distance between these dialects and the standard Dutch varieties for which ASR tools have already been developed, and (iv) the high transcription accuracy needed for the further linguistic annotation and analysis of the dialect data. Of course, once the corpus is available, the transcripts can be used to train new dialectal/regiolectal recognizers of Dutch.

### Respeaking

As discussed above, quality requirements for dialect transcriptions can at present often not be met by state-of the-art ASR technology. There are however other alternatives to a purely manual transcription approach, combining human skill, and speech technology. Sperber et al. (2013), for instance, suggest *respeaking* to provide a good trade-off between transcription quality and cost. In *respeaking*, a speaker repeats and records the speech of the original speaker using a speech recognition system. *Respeaking* is assumed to be faster than typing, and allows circumventing some of the problems in ‘pure’ ASR approaches, as the respeaker's voice can be recorded in a strictly controlled setting (cf. sound quality problem discussed above) and the ASR system can be trained or adapted to the voice of the respeaker.

Respeaking is nowadays often used to (i) subtitle live broadcasts (cf. Imai et al., 2002; van Waes et al., 2013), typically when there is no script available (Romero-Fresco, 2011) or (ii) to lower the cost of speech transcription via crowd-powered speech transcription platforms (cf. Vashistha et al., 2017). Of course, as respeaking partly builds on ASR tools, it is also sensitive to errors. Therefore, an editor or the respeaker often manually corrects the initial draft transcription (van Waes et al., 2013, p. 18) or ASR transcripts of the same audio respoken by multiple respeakers are compared and combined (Vashistha et al., 2017).

Respeaking also has applications in linguistic research and, in fact, in a dialect corpus project somewhat similar to the GCND. For the Spanish COSER corpus, a respeaker approach is adopted to build a parsed corpus of European Spanish dialects (Rufino Morales, 2019). One respeaker from Granada, who understands most peninsular Spanish dialects well, has been trained to respeak interviews made between 1990 and now.

By way of trial, one of the authors of this paper—a variationist linguist and native speaker of the West Flemish dialect—respoke the excerpt in Example (3), standardizing non-standard vocalism. The resulting audio was then fed into the ASR tool of the BASWebServices of the Ludwig Maximilian University of Munich.^23^ The WER of the resulting transcript [see (6), with, for the sake of convenience, also a repetition of the manual reference transcription in (7)]−34%—is remarkably lower than the one obtained by applying ASR on the original audio (95%). Thirty-four percent is still high—as already discussed at the end of the previous section a WER of this size still requires too much manual correction to be useful—but it might be seen as a sign that with the necessary training and technical optimization, respeaking could be a valuable technique in the transcription process.

(6) k intern qua verankerd in de fabriek van een van mevrouw drieëntwintig tot negenentwintig en in nee op het laatste zijn hier komen werken in de file vierendertig jaar en k moeten twee jaar eerder mijn pensioen nemen onderdak pakt waren aan mijn hart en zo ik zijn nu gepensioneerd zijn nu tweeënzestig nu nieuwjaar twee dagen voor het nieuwe jaar zei tweeënzestig ja en ik zijn al elf jaar mijn man kwijt wil ja ik ga maar één
I internal qua anchored in the factory of a of madam twenty-three to twenty-nine and in no at the end am here come work in the traffic-jam thirty-four year and I have to two year earlier my retirement am now sixty-two now new year two days before the new year said sixty-two yes and I am already eleven year my husband lost want yes I go but one
(7) k#e vijf jaar in Tourcoing ewrocht in e fabrieke. van negen…uh van drieëntwintig tot negenentwintig. en in ne…op t#laatste van negenentwintig k#zijn ier komen werken in de filature. vierendertig jaar. en k#e moeten twee jaar eerder mijn pensioen nemen. omda#k epakt waren aan mijn harte. en ezo k#zijn nu gepensioneerd. k#zijn nu tweeënzestig nu nieuwjaar. twee dagen voor nieuwjaar zij#k tweeënzestig. ja en k#zijn al elf jaar mijn man kwijt wi. awel ja#k. ja k#e maar een zoone.
*I have worked for five years in Tourcoing in a factory. From nine… uh from twenty-three to twenty-nine. and in… in the end from twenty-nine I have come here to work in the filature. thirty-four years. and I have had to retire two years earlier. because I had heart problems. and as such I am retired now. I'll turn sixty-two at new year. two days before New Year I am sixty-two. yes and I have lost my husband for eleven years already. yes I have. yes. I have only one son*.

There are, however, a number of issues to bear in mind, in particular with respect to projects like the current one. Firstly, the respeaker must understand the dialect(s) well. In the case of the COSER corpus, the respeaker from Granada is able to cover a lot of the Iberian Peninsula, but in other language communities and also when it concerns older recordings, affected less by dialect leveling, such wide intelligibility is everything but self-evident (cf. Boberg et al., 2018, p. 5 on mutual intelligibility of dialects and clines of linguistic similarity). The (southern) Dutch dialects for instance, as they are recorded in the *Stemmen uit het Verleden* collection, display significant linguistic differences between each other, as well as with the standard language, on which the tools are trained. As stated earlier, these differences also concern such typological traits as word order and inflectional morphology. Cliticization and pronoun doubling are cases in point. In (8), five clitics form a cluster that behaves like one phonological word. In order to transcribe such a sequence adequately using respeaking, separate pronunciation on the part of the respeaker is required. This would require the respeaker to parse such clitic clusters in real time.

(8) Recording H68_Loppem
                   k#en#e#k#ik nooit niet gezien
                   ik en heb ik ik nooit niets gezien
                   I NEG have I I never nothing seen
                   “I have never seen anything.”

The historical SDDs in the collection already show significant typological differences already within a short geographical distance. As it is highly unlikely that one could find a single respeaker capable of understanding all these dialects, multiple respeakers [e.g., (at least) one per dialect region] would have to be trained for the GCND. This implies that the ASR software would also have to be trained for multiple speakers. Evidently, the time and money needed to (a) train these respeakers, (b) (re)train the ASR systems, and (c) correct the draft transcripts is not sufficiently compensated by the gain in time respeaking is said to have over typing. Secondly, respeaking requires quickness of response to the original audio (Romero-Fresco, 2011). In the case of the GCND audio collection, which actually represents historical speech, transcribers often consult dialect dictionaries and studies on local customs and folklore to determine what the dialect speakers in the recordings are talking about. This of course complicates the respeaking process. Thirdly, respeaking is also sensitive to some of the problems encountered when discussing ASR (cf. section Automatic speech recognition), e.g., the training data needed to adjust the ASR system. The advantage of respeaking is that the respeaker can standardize dialectal pronunciations of standard language words, but of course (i) such standardization requires a serious cognitive effort and (ii) the respeaking system still has to be able to handle dialectal lexemes (especially when the goal is to build a dialect corpus). At the same time, certain morphological, syntactic, and lexical phenomena should in fact not be standardized, as argued above. For all these reasons, the decision was made not to use respeaking in the GCND project.

### Forced Alignment for Automatic Segmentation and Phonetic Transcription

Another alternative to ‘pure’ ASR that combines speech technology with human effort is FA, the process of aligning speech (audio) with text (the written representation of the recorded speech). FA requires transcriptions as input (made manually or automatically), and as such does not clear the transcription hurdle. It does, however, allow (i) automatically creating phonetic transcriptions on the basis of orthographic ones and (ii) automatically aligning the text transcription to the audio on a word or phoneme level (the latter is also called phonetic alignment).

**Figure 3:**
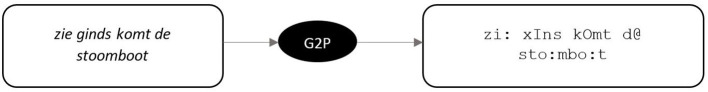
Grapheme-to-phoneme conversion of the Dutch sentence *Zie ginds komt de stoomboot* (‘see the steamboat over there’).

In FA, the input text is parsed into a chain of words and subsequently passed to a grapheme-to-phoneme (G2P) algorithm (cf. Figure 3), which results in a string of phonemic symbols.^24^ As a rule, this happens via the canonical transcriptions of the words in the text, i.e., the way in which—according to some predefined standard (either specifying the pronunciation rules of a language or combining a lexical pronunciation dictionary with fallback to the rule-based system)—the words ought to be pronounced. More advanced G2P algorithms also take into account phonetic processes that occur when combining certain words (e.g., assimilation) or pronunciation variants that may occur in spontaneous speech [cf. WORDVAR in the Munich Automatic Segmentation (MAUS) system, (Schiel, 1999)], but nonetheless, the phonetic rendering is always based on how the words in the text are expected to be pronounced on the basis of a defined standard or system, not on how the speaker has actually pronounced these words.

**Figure 4:**
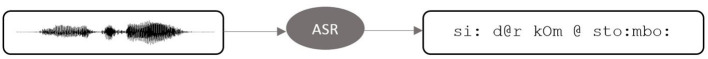
Automatic speech recognition (from audio to transcription).

Parallel to the G2P conversion, the speech signal is transcribed phonetically by means of ASR (cf. Figure 4 and the earlier section on automatic speech recognition). In the case of the example in Figures 3, 4, the pronunciation of the speaker, as ‘decoded’ by ASR, does not entirely match the canonical transcription made on the basis of the input text (e.g., with devoicing of the /z/ in the word *zie* in the speech signal).

A next step consists of aligning the outputs of both G2P and ASR (the actual FA), attempting to match the two sequences as ‘efficient’ as possible. In Table 1, gray cells represent phonemes where there is a match between the two outputs, yellow cells involve substitutions and red cells indicate that an ‘expected’ sound is not detected in the actual speech signal.

**Table 1:** Forced alignment of G2P and ASR output.

**Input text**	***zie***		***ginds***				***komt***				***de***		***stoomboot***						
G2P output	z	i:	x	I	n	s	k	O	m	t	d	@	s	t	o:	m	b	o:	t
ASR output	s	i:	d	@	r		k	O	m			@	s	t	o:	m	b	o:	
Speech	*T1*	*T2*	*T3*	*T4*	*T5*	*T6*	*T7*	*T8*	*T9*	*T10*	*T11*	*T12*	*T13*	*T14*	*T15*	*T16*	*T17*	*T18*	*T19*

As the speech recognizer determines begin and end times for each of the detected sounds, it is possible to calculate the begin and end times of the words, even when the ‘dictionary’ pronunciation does not (entirely) match the actual pronunciation. As such, the text transcriptions can be linked to the audio on a phoneme and word level, allowing researchers interested in the pronunciation of specific words or sounds to find these more easily in a speech corpus and to export the relevant portions of the audio efficiently into speech analysis software (such as Boersma and Weenink, 2011). However, the accuracy of the time codes does decrease inversely proportional to the differences between the norm pronunciation and the actual pronunciation.

Some FA applications also allow automatic phonetic transcription. The Munich Automatic Segmentation system (Schiel, 1999) for instance generates, on the basis of the canonical phonetic transcription of an orthographic transcription fed into the system, an acyclic-directed graph of all probable pronunciation variants of the input utterance, along with the predictor probability of these variants. Subsequently, the graph and the speech wave are “passed to a standard Viterbi alignment procedure that computes the best combined probability of acoustical score and predictor probability, in other words, finds the most likely path through the graph” (Schiel, 1999, p. 2). As such, a (broad) phonetic transcript is created that combines information from (i) the speech signal (the actual speech), (ii) an orthographic transcription, and (iii) specified knowledge about the pronunciation of a certain language.

FA has many applications in linguistic research. The corpus of spoken Dutch (CGN) for instance applied FA not only to align the speech signal at word level to the orthographic transcription but also to automatically generate broad phonetic transcriptions of about 900 h of recorded speech on the basis of orthographic transcriptions. Goddijn and Binnenpoorte (2003) report error rates ranging from 15% for spontaneous speech to 6% for read speech and conclude that automatic phonetic transcription on the basis of orthographic transcripts is the best approach for their spoken (near-)standard Dutch data, in combination with manual correction. The inverse procedure is also possible: creating an orthographic transcription departing from a phonetic one. In the Nordic Dialect Corpus for instance, all Norwegian dialects and some Swedish ones were first transcribed phonetically, and subsequently, the phonetic transcriptions were translated to orthographic ones via a semi-automatic dialect transliterator developed for the project (Johannessen et al., 2009). Of course, manual phonetic transcription is more time-consuming than manual orthographic transcription. Another application of FA can be found in the automatic extraction of variables for phonetic analysis (cf. Evanini et al., 2009 and Rosenfelder et al., 2014 on the FAVE automated vowel extraction program and Reddy and Stanford, 2015 on DARLA, which automatically generates transcriptions with ASR and extracts vowels using FAVE).

**Figure 5:**
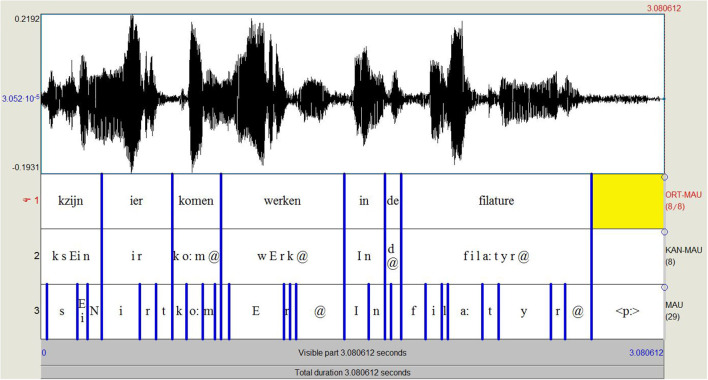
WebMAUS output for the dialect sentence *kzijn ier komen werken in de filature* (‘I have started to work here in the filature’).

Figure 5 shows the output of a FA test using the BASWebServices of the Ludwig Maximilian University of Munich (Schiel, 1999; Kisler et al., 2017).^25^ Their WebMAUS-module segments an audio file into SAMPA phonetic segments given an orthographic transcription. We fed the dialect sentence *kzijn ier komen werken in de filature* [cf. Example (2) above] with the corresponding audio into WebMAUS, selecting as language ‘Dutch (BE).’ The first layer (‘ORT-MAU’) shows the original orthographic transcription (following the project protocol). The second layer (‘KAN-MAU’) represents the canonical phonetic transcriptions created by the G2P algorithm on the basis of ‘Dutch_BE’ as specified language, and the third layer (‘MAU’) shows the automatic phonetic transcription, representing the best combined probability of acoustical score and predictor probability (cf. supra).

We subsequently tested the accuracy of the WebMAUS aligner on a slightly longer stretch of West Flemish dialect speech [Example (3)]. The word boundaries determined by the forced aligner are remarkably accurate: 81% of the 90 words are accurately delimited, notwithstanding the fact that the pronunciation of the speaker deviates clearly from the standard Dutch pronunciation of the words used. The phonetic transcription and delineation of phonemes (cf. layer 3 in Figure 5) are a bit less accurate, but still good. We obtain a phoneme error rate^26^ of 28%, which is not perfect—it certainly is not good enough to use for phonetic research without manual correction—but it is also not disastrous, especially considering the absence of acoustic, and language models for the SDDs. The automatic phonetic segmentation and transcription could be improved, by either (i) feeding phonetic transcriptions into the system, making it possible to skip the G2P procedure or—less ideal, but more feasible—(ii) departing from an orthographic transcription that renders more of the pronunciation peculiarities in the text than our current transcriptions do (‘eye dialect transcription’). Concerning (i), we already indicated that phonetic transcriptions are too time-consuming and too prone to intertranscriber inconsistencies. Concerning (ii), fixed rules in the dialect (e.g., <ij> in orthography should be pronounced as [i] in many West Flemish dialects) can be specified to automatically add pronunciation information in existing transcriptions. In the case of the SDDs, however, ‘dialect rules’ often depend from place to place and are in many cases also lexically diffuse (meaning that a rule applies to some words, but not to others). It is hence difficult to list ‘dialect rules’ that apply to all words with a specific orthography in all SDDs. As an alternative, we deliberated adjusting the transcription protocol in such way that the first layer of the transcription (closest to the dialect) would be more of an ‘eye dialect’ rendering than was the case in the SAND protocol from which we departed. This can be seen as a middle course between full phonetic transcription and a more standardized orthographic transcription, which might improve the automatically generated phonetic transcriptions. We therefore retested the WebMAUS FA on Example (3), now with an orthographic transcription that marked more dialectal pronunciations. In this new transcription, we, for instance, wrote *zin* and *kwit* instead of *zijn* (‘to be’) and *kwijt* (‘lost’) to indicate that the old West Germanic î is realized as a monophthong [i] before non-labiodentals in many West Flemish dialects. The new transcription also marked (i) schwa-deletions (e.g., by writing *moetn* instead of *moeten* ‘have to’), (ii) h-deletions (*erte* instead of *harte* ‘heart’), (iii) the shortening of [aː] to [ɑ] (*latste* instead of *laatste* ‘last’), (iv) the palatalization of [oː] in certain words (e.g., *zeune* instead of *zoone* ‘son’), (v) the velarization of [aː] to [ɔː] (e.g., *joar* instead of *jaar* ‘year’), and (vi) the realization of an intervocalic [j] in words such as *drie(j)ëntwintig* (‘twenty-three’). Fed into the FA system, this adapted orthographic transcription did not improve the word segmentation success (now 79% of the 91 words were correctly delineated), but it did cause a decrease of 5% in the phoneme error rate (resulting in an error rate of 23%).

Our test results indicate that FA can be very useful for dialect corpus building. In the context of the GCND corpus, we decided to apply FA for word-level segmentation. This word-level segmentation is interesting as it allows searching for and extracting the pronunciation of individual words in the corpus, useful in, for instance, lexical, and phonological projects. Phonological/phonetic research is not the primary goal of the corpus project, but all the same the intention is to make the corpus as multi-usable as possible. Word-level segmentation also allows a detailed alignment of word-level annotations (such as POS tags) to the audio. Given the low error rates the aligner obtained with our data, it seems possible to apply word-level alignment without much manual correction. Manual correction is however clearly needed when FA is applied for automatic phonetic transcription. FA can certainly speed up the transcription process by providing a rough first transcription as a starting point, but to make this useful for phonetic research, a serious time investment is still needed. For the GCND project, the decision was therefore made not to invest in FA for phonetic transcription. Phoneticians interested in the corpus can, however, of course apply FA themselves to create phonetic transcriptions. We also decided not to alter the original transcription protocol in the direction of a more ‘eye dialectal’ rendering of non-standard vocalism, as the improvement this rendering brought for FA was in our opinion too small to compensate for the extra complexity eye dialect renderings add to the manual transcription process. Hence, the decision was made to stick with the original transcription protocol, as this guaranteed more consistency among transcribers.

## Transcription Procedure in the GCND

After weighing the advantages and disadvantages of existing speech technological tools for the transcription of dialectal speech, the decision was made to manually transcribe the dialect recordings of the ‘Voices from the Past’ collection in two layers, each aligned to the audio at sentence level using the software package ELAN (Max Planck Institute for Psycholinguistics, cf. Brugman and Russel, 2004). This manual transcription is very time-consuming—with transcription speeds for our data ranging from 67 s/h for a beginning transcriber to 120 s/h for an experienced transcriber—but it is at the moment still the most efficient option, as ASR has much difficulties handling the SDDs and as such yields transcriptions with error rates that are too high to be useful for linguistic research. Speech technology, and more specifically FA, can however be helpful to automatically refine the rough manual alignment of the transcription to the audio (which happens at sentence or clause level) to a word-level alignment, facilitating phonetic research.

A difficult question in the GCND project was what to do with the existing 318 transcriptions, which—as mentioned in section Toward a corpus of Southern Dutch Dialects —are currently only available in the form of scans (i.e., image files) of the original typewritten or even handwritten texts. It is of course possible to use optical character recognition (OCR) on these image files and have a forced aligner align the resultant text files to the audio file, but the problem remains that the transcriptions are very heterogeneous in the way the dialect has been transcribed orthographically (cf. Figure 2) and that the original transcriptions often contain many mistakes. Also considering that the OCR and FA procedures would cause extra mistakes (given the diversity in input image files, cf. Figure 2), manual editing would still be necessary, adjusting the texts to the new protocol, adding a second layer of transcription, and correcting mistakes of both the transcriber, the OCR and the forced aligner, which raises the question whether it is not more time-efficient to make a new (manual) transcription from scratch, using the original transcription as resource to speed up the transcription process. The decision was made to not invest time in optimizing and executing OCR and FA procedures, as manual labor was necessary anyhow.

Of vital importance when working with human transcribers is that a detailed, yet workable transcription protocol is developed and that sufficient training is provided. For the GCND project, five student-transcribers^27^ tested a first version of the protocol described in the section ‘Building on existing standards’. They were asked to keep a log of problems they encountered during transcribing, which was subsequently discussed during weekly group meetings with the project leaders. During this test phase, the protocol was refined and elaborated with examples. A next group of 15 student-transcribers was hired and trained to work with the new protocol. To guarantee transcription accuracy and consistency, all students received (i) a group demo of the software and the protocol, (ii) online training materials, (iii) personalized feedback on their initial transcriptions (random samples were corrected by the project supervisors), and (iv) access to a shared ‘problem database,’ where dubious cases could be registered and the project supervisors subsequently offered advice on how to transcribe the problematic utterance in line with the protocol.

Of course, human transcribers are also not infallible. To guarantee the quality of the transcriptions, a crowd-sourcing network has been established in which volunteers check the transcriptions made by student-transcribers. These volunteers especially focus on speech fragments marked with the code “???” by the transcribers. The ??? code indicates passages that the student-transcribers did not understand, either because of gaps in their dialect proficiency or because of limited familiarity with the speech topic (e.g., when the interviewee talks about farming techniques or barrel making).^28^ Contrary to the student-transcribers, most volunteers acquired the traditional dialect as a first language. They generally also have more life experience—the majority of volunteers have retired—and are hence usually more tuned in to the subject matter than the student-transcribers. The volunteers check the accuracy of the transcriptions on paper or text files exported from ELAN; their corrections and additions are evaluated and adjusted in ELAN by a project worker fully acquainted with the protocol and the software. As already mentioned in the section on Automatic speech recognition, comparison of initial student transcriptions with the final, corrected equivalents for four recordings of four different dialect areas yields an average WER of 2.93%, which, in comparison with the WERs of ASR tools, is very low and argues in favor of manual transcription.

## Conclusions and Recommendations for Practice

There are at present many speech technological tools available that can speed up the transcription of spontaneous speech, such as ASR, respeaking, and FA, but dialects—at least when defined in the ‘traditional’ sense as a regionally determined language varieties which differ at multiple structural levels from other dialects and the ‘overarching’ standard language—still constitute a major challenge. For the transcription of the dialect audio collection available at Ghent University (*Stemmen uit het Verleden* ‘Voices from the past’), the choice was made to use speech technology only for the word-level segmentation of the audio files, as the transcription itself could not be sped up by ASR tools. This decision is however not necessarily also appropriate for other dialect corpus projects. In deliberating the usefulness of speech technological tools for a dialect corpus project, the following questions have to be considered:

- **What is the sound quality of the recordings?** If the recording quality is high (with a high-quality external microphone, little background noise, or overlapping speech and a similar distance to the microphone for all speakers), speech technological tools should be considered. Recordings of poorer quality, however, with more interference and more heterogeneous speech, still pose a major challenge for speech technological tools such as ASR, particularly in the absence of suitable models. This problem can, when the conditions discussed below are favorable, be circumvented using respeaking. As respeaking combines ASR with human ‘labor’—a respeaker repeats and records the speech of the original speaker using a speech recognition system—poor audio quality or heterogeneity of the original speech can be set right in the first step of the respeaking process.
- **Which resources are available for the dialect(s) under study?** Application of ASR can be considered if a pronunciation dictionary for the dialect(s) has been developed, or—even better—if acoustic and language models are available for the dialect(s) and/or overarching standard language. When pronunciation dictionaries or acoustic or language models are only available for the standard language, and not for the dialect(s) under study, the usefulness of speech technological tools strongly depends on the way in which standard and dialect(s) differ.
- **What is the degree of linguistic differentiation between the dialects in the corpus and the standard language?** If the distance between the dialects is large and no straightforward rules can be formulated about the correspondences between these dialects (e.g., sound X in dialect A always corresponds to sound Y in dialect B or in the standard language, cf. Rys and Van Keymeulen, 2009), multiple recognition systems have to be built for ASR (or tools integrating ASR, such as FA), implying serious time and financial investments. If the distances, however, are small, or systematic correspondence rules can be listed for the differences between the dialects or between the dialects and the standard language, it can be considered to develop dialect-specific acoustic and language models for ASR tools. Linguistic differentiation is also an important criterion when considering the usefulness of respeaking. If the dialects under study are mutually intelligible, one respeaker can be trained to handle the whole dataset. If the dialects are not or only partially mutually intelligible, respeaking poses a bigger challenge.
- **Which goals do the transcripts have to serve?** If the main goal is to make recordings searchable in terms of content, a moderate transcription accuracy (with WERs up to 50%) is often perfectly acceptable, and such accuracy can be achieved using speech technology. If the transcripts, however, have to serve as input for linguistic research, a higher transcription accuracy is needed. In that case, the researcher has to weigh the advantages of a procedure consisting of ASR [with or without respeaker(s)] and subsequent manual correction against those of manually transcribing the recordings from the beginning. Also, it should be considered which type of linguistic research the transcripts have to facilitate. If the focus is mainly on syntax, lexicon, or morphology, an orthographic transcription of the original audio suffices and word-level alignment of the audio to the transcription is perfect. Such word-level alignment can be perfectly achieved—in case one ultimately decides not to transcribe with ASR from scratch—with the help of FA. If phonetic research is intended, FA can also automatically generate phonetic transcriptions on the basis of orthographic ones. Manual correction is still needed, but the broad phonetic transcription created by FA can speed up the phonetic transcription process (cf. above).

Only when all of these questions have been addressed is it possible to decide whether or not to invest in ASR development for dialect transcription. In case the deliberation militates in favor of manual transcription, it is important that a detailed protocol is developed and tested in interaction with multiple transcribers and that sufficient attention is paid to the training of transcribers, with the necessary opportunities for feedback.

In all probability, significant headway will in the next few years be made in the automatic recognition of non-standard speech. While the interests of computational linguists and dialectologists might diverge at some points—as dialect shift and leveling processes progress, the dialects in the ‘Voices from the past’ collection for instance increasingly represent a historical stage of the language, which is greatly interesting for linguists modeling theories on language variation and change, but might appeal less to computational linguists training speech recognizers to handle everyday speech—cooperation between dialectologists and ASR specialists is undoubtedly fruitful. Speech recordings transcribed and annotated manually by dialectologists are useful training materials for computational linguists, even when the dialects represent the language of only a fraction of a speech community. In diglossic communities for instance (Auer, 2005), where a continuum of intermediate varieties has developed between the traditional dialects and the official standard language (e.g., in Dutch-speaking Belgium or Germany), intermediate varieties are generally marked by a combination of dialect and standard language variants. In such contexts, a speech recognizer that can handle both local dialects *and* standard language can handle a large part of the sociolinguistic repertoire. To be continued…

## Data Availability Statement

The dialect recordings discussed in this contribution can be consulted freely on www.dialectloket.be.

## Ethics Statement

The studies involving human participants were reviewed and approved by the Ethics Committee of the Faculty of Arts and Humanities at Ghent University. Written informed consent for participation was not required for this study in accordance with the national legislation and the institutional requirements.

## Author Contributions

JV, AB, and A-SG devised the project. AB was in charge of overall direction and planning. A-SG developed the transcription protocol with help from JV. MF contributed transcriptions to the project, was—together with A-SG and AB—responsible for the training of the student-transcribers, and computed the WERs of the manual transcription procedure. A-SG performed the tests discussed in the sections on speech technological tools, under the guidance of AH with input from all authors. A-SG wrote the manuscript.

### Conflict of Interest

The authors declare that the research was conducted in the absence of any commercial or financial relationships that could be construed as a potential conflict of interest.

## Footnotes

^1^In this paper, we clearly distinguish between *dialect* and *accent*, regarding accent as “restricted to phonological and especially phonetic differences, such as the quality of vowel sounds” (Boberg et al., 2018, p. 4).

^2^Debate is possible on the exact delineation of the SDDs (cf. Taeldeman and Hinskens, 2013); for reasons of comparability, the corpus project described in this paper will span the same geographical area as other major dialectological projects (cf. e.g., Van Keymeulen et al., 2019).

^3^In the near future, we hope to expand the corpus, also by collaborating with the Meertens Institute, to include recordings from their dialect database (the so-called *Nederlandse Dialectenbank*, ‘Dutch Dialect Bank’).

^4^The standard language is mainly based on the sociolect of the middle class in the cities of the provinces North- and South-Holland (see Willemyns, 2003 for a historical background).

^5^Cf. the ‘comment’ section in the data from the surveys on http://www.meertens.knaw.nl/sand/zoeken/lijst_met_plaatsen.php

^6^These tags indicate the grammatical category (e.g., noun, adverb, or preposition) of each word in the text.

^7^COSER = *Corpus Oral y Sonoro del Español Rural* (‘Audible Corpus of Spoken Rural Spanish’). http://corpusrural.es

^8^PRESEEA = *Proyecto para el Estudio Sociolingü*í*stico del Español de Españay de América* (‘Project for the Sociolinguistic Study of Spanish from Spain and America’). http://preseea.linguas.net/Corpus.aspx

^9^FOLK = *Forschungs- und Lehrkorpus Gesprochenes Deutsch* (‘Research and Teaching Corpus of Spoken German’). http://agd.ids-mannheim.de/folk.shtml

^10^CORDIAL-SIN = *Corpus Dialectal para o Estudo da Sintaxe* (‘Syntax-oriented Corpus of Portuguese Dialects’). https://clul.ulisboa.pt/en/recurso/cordial-sin-syntax-oriented-corpus-portuguese-dialects

^11^https://dioe.at/

^12^https://clul.ulisboa.pt/sites/default/files/inline-files/manual_normas.pdf

^13^In the CGN project, transcribers were instructed to use words from a predefined lexicon, which contained (in principle) all Standard Dutch words and also a number of commonly occurring reduced forms. Dialect words or constructions not occurring in Standard Dutch (and hence also not in the CGN lexicon) are marked with the label ‘^*^d’, whereas standard Dutch words pronounced in a “heavily dialectal way” get the label ‘^*^z’. Such an approach is not feasible for a dialect corpus project, as about any word, word form, or construction would have to be marked by either ‘^*^d’ or ‘^*^z’.

^14^These data are now freely available online via http://www.meertens.knaw.nl/sand/zoeken/. With the MIMORE tool (http://www.meertens.knaw.nl/mimore/search/), the researcher can search in the *Dynamic Syntactic Atlas of the Dutch Dialects* combined with two other databases (*Diversity in Dutch DP* and the *Goeman, Taeldeman, van Reenen Project*) with a common online search engine. The search results can be visualized on geographic maps and exported for statistical analysis.

^15^Can be consulted via www.woordenlijst.org.

^16^https://tla.mpi.nl/tools/tla-tools/elan/

^17^http://innovatieveoverheidsopdrachten.be/projecten/spraaktechnologie-voor-verslaggeving-vlaams-parlement

^18^The WER is “the edit distance between a reference word sequence and its automatic transcription, normalized by the length of the reference word sequence” (McCowan et al., 2005, 2): WER = (S+D+I)/N_r_, with N_r_ as the total words in the reference transcription, S as the number of substituted words in the automatic transcription, D as the number of words from the reference transcription deleted in the automatic transcription, and I as the number of words inserted in the automatic transcription not appearing in the reference. See McCowan et al. (2005) for a critical discussion.

^19^A short excerpt (89 words) was selected from the dialect recording of Ieper (West Flanders).

^20^https://clarin.phonetik.uni-muenchen.de/BASWebServices/interface, language selected: “Dutch (Netherlands) – conversation.”

^21^Words such as *daskapan* also make no sense in Dutch.

^22^Adjusted to standard Dutch spelling systems and regularizing dialectal pronunciation features. The West Flemish word [ɦəbø:rne:ɦə] is for instance written down as *gebuurnege* (Standard Dutch *buurvrouw* ‘female neighbor’), in which the West Flemish laryngalisation of standard Dutch /ɤ/ and the West Flemish realization of West Germanic û before /r/ as [ø˙] are standardized and written down as < g> and < uu> (cf. standard Dutch [ɤ] and [y˙]), respectively.

^23^https://clarin.phonetik.uni-muenchen.de/BASWebServices/interface, language selected: “Dutch (Netherlands) – conversation.”

^24^Phonemes are generally written down in SAMPA (Speech Assessment Methods Phonetic Alphabet), which is a computer-readable phonetic script using 7-bit printable ASCII characters, based on the International Phonetic Alphabet (IPA).

^25^Cf. https://clarin.phonetik.uni-muenchen.de/BASWebServices/interface/WebMAUSBasic

^26^The Phoneme Error Rate was calculated in a similar way to the word error rate: (S+D+I)/Nr, with Nr as the total number of phonemes in the reference transcription, S as the number of substituted phonemes in the automatic transcription, D as the number of phonemes from the reference transcription deleted in the automatic transcription, and I as the number of phonemes inserted in the automatic transcription not appearing in the reference.

^27^All transcribers working in the pilot project were students in linguistics at Ghent University.

^28^Fragments that were difficult to transcribe for acoustic reasons (e.g., recording quality, background noise or overlapping speech) are marked with a different code (“xxx”).
